# Eukaryotic genomes from a global metagenomic data set illuminate trophic modes and biogeography of ocean plankton

**DOI:** 10.1128/mbio.01676-23

**Published:** 2023-11-10

**Authors:** Harriet Alexander, Sarah K. Hu, Arianna I. Krinos, Maria Pachiadaki, Benjamin J. Tully, Christopher J. Neely, Taylor Reiter

**Affiliations:** 1 Biology Department, Woods Hole Oceanographic Institution, Woods Hole, Massachusetts, USA; 2 Marine Chemistry and Geochemistry, Woods Hole Oceanographic Institution, Woods Hole, Massachusetts, USA; 3 MIT-WHOI Joint Program in Oceanography/Applied Ocean Science and Engineering, Cambridge and Woods Hole, Massachusetts, USA; 4 Department of Biological Sciences, University of Southern California, Los Angeles, California, USA; 5 Department of Quantitative and Computational Biology, University of Southern California, Los Angeles, California, USA; 6 Population Health and Reproduction, University of California, Davis, Davis, California, USA; University of Maryland School of Medicine, Baltimore, Maryland, USA

**Keywords:** metagenomics, protists, genomes, eukaryotic metagenome-assembled genomes

## Abstract

Metagenomics is a powerful method for interpreting the ecological roles and physiological capabilities of mixed microbial communities. Yet, many tools for processing metagenomic data are neither designed to consider eukaryotes nor are they built for an increasing amount of sequence data. EukHeist is an automated pipeline to retrieve eukaryotic and prokaryotic metagenome-assembled genomes (MAGs) from large-scale metagenomic sequence data sets. We developed the EukHeist workflow to specifically process large amounts of both metagenomic and/or metatranscriptomic sequence data in an automated and reproducible fashion. Here, we applied EukHeist to the large-size fraction data (0.8–2,000 µm) from Tara Oceans to recover both eukaryotic and prokaryotic MAGs, which we refer to as TOPAZ (Tara Oceans Particle-Associated MAGs). The TOPAZ MAGs consisted of >900 environmentally relevant eukaryotic MAGs and >4,000 bacterial and archaeal MAGs. The bacterial and archaeal TOPAZ MAGs expand upon the phylogenetic diversity of likely particle- and host-associated taxa. We use these MAGs to demonstrate an approach to infer the putative trophic mode of the recovered eukaryotic MAGs. We also identify ecological cohorts of co-occurring MAGs, which are driven by specific environmental factors and putative host-microbe associations. These data together add to a number of growing resources of environmentally relevant eukaryotic genomic information. Complementary and expanded databases of MAGs, such as those provided through scalable pipelines like EukHeist, stand to advance our understanding of eukaryotic diversity through increased coverage of genomic representatives across the tree of life.

**IMPORTANCE:**

Single-celled eukaryotes play ecologically significant roles in the marine environment, yet fundamental questions about their biodiversity, ecological function, and interactions remain. Environmental sequencing enables researchers to document naturally occurring protistan communities, without culturing bias, yet metagenomic and metatranscriptomic sequencing approaches cannot separate individual species from communities. To more completely capture the genomic content of mixed protistan populations, we can create bins of sequences that represent the same organism (metagenome-assembled genomes [MAGs]). We developed the EukHeist pipeline, which automates the binning of population-level eukaryotic and prokaryotic genomes from metagenomic reads. We show exciting insight into what protistan communities are present and their trophic roles in the ocean. Scalable computational tools, like EukHeist, may accelerate the identification of meaningful genetic signatures from large data sets and complement researchers’ efforts to leverage MAG databases for addressing ecological questions, resolving evolutionary relationships, and discovering potentially novel biodiversity.

## INTRODUCTION

Unicellular microbial eukaryotes, or protists, play a critical part in many ecosystems found on the planet. In addition to their vast morphological and taxonomic diversity, protists exhibit a range of functional roles and trophic strategies (1). Protists are central to global biogeochemical cycles, mediating the pathways for the synthesis and processing of carbon and nutrients in the environment (2–4). Despite their importance across ecosystems and in the global carbon cycle, research on microbial eukaryotes typically lags behind that of bacteria and archaea (5, 6). Consequently, fundamental questions surrounding microbial eukaryotic ecological function *in situ* remain unresolved. Novel approaches that enable genome retrieval from meta’omic data provide a means of bridging that knowledge gap.

Assembled genetic fragments (derived from metagenomic reads) can be grouped together based on their abundances, co-occurrences, and tetranucleotide frequency to reconstruct likely genomic collections, often called bins (7–10). These bins can be further refined through a series of steps to ultimately represent metagenome-assembled genomes or MAGs (11–14). Binning metagenomic data into MAGs has revolutionized how researchers ask questions about microbial communities and enabled the identification of novel bacterial and archaeal taxa and functional traits (15, 16), but only recently has the recovery of eukaryotic MAGs become more common (17–19). The reason for the differential recovery between prokaryotic and eukaryotic MAGs is arguably twofold: (i) eukaryotic genomic complexity (20) complicates both metagenome assembly and MAG retrieval, and (ii) there is a bias in currently available metagenomic computational tools toward the study of bacterial and archaeal members of the community. Continued computational efforts to expand and enhance the recovery of eukaryotic genomic information through reproducible workflows and pipelines will help us resolve questions surrounding the evolutionary relatedness and population genetics of the unculturable majority of eukaryotic microbes.

Here, we developed and applied EukHeist, a scalable and reproducible pipeline to facilitate the reconstruction, taxonomic assignment, and annotation of prokaryotic and eukaryotic MAGs from mixed community metagenomes. The EukHeist pipeline incorporates metagenome reads to first generate all environmentally relevant MAGs and then sort putative eukaryotic MAGs from bacteria and archaea; the easily customizable workflow can also include metatranscriptome reads to investigate transcriptionally active portions of MAGs. To demonstrate the scalability and utility of EukHeist across a large data set, we applied it to the Tara Oceans expedition protist-size fractions’ samples (21), which encompasses more than 20 TB of raw sequence data. Our multi-domain approach for MAG retrieval of mixed microbial communities recovered over 4,000 prokaryotic MAGs and 900 eukaryotic MAGs. We explore how genome length, *in situ* microbial diversity, ocean region, and depth influence the resolution of MAGs and use highly complete eukaryotic MAGs to better understand microbial eukaryotic trophic modes and how environmental factors influence the co-occurrence of marine eukaryotes and prokaryotes. The application of EukHeist and our results highlight the value of using large, untargeted approaches in exploring environmentally relevant genomic signatures in nature.

## RESULTS AND DISCUSSION

The EukHeist metagenomic pipeline was designed to automate the recovery and classification of eukaryotic and prokaryotic MAGs from large-scale environmental metagenomic data sets. EukHeist was applied to the metagenomic data from the large-size fraction metagenomic samples (0.8–2,000 µm) from Tara Oceans (21), which is dominated by eukaryotic organisms. We generated 94 co-assembled metagenomes based on the ocean region (OR), size fraction (SF), and depth (D) of the samples (Fig. S1), which totaled 180 Gbp in length (Table S1). A total of 988 eukaryotic MAGs and 4,022 prokaryotic MAGs were recovered; these MAGs have been made available under the name Tara Oceans Particle-Associated MAGs, or TOPAZ (Tables S2 and S3, available through open science framework at https://osf.io/gm564/). The TOPAZ MAGs expand the current repertoire of publicly available eukaryotic genomic references for the marine environment and complement other efforts to recover eukaryotic MAGs from the same large size-fraction data set (17). Here, we highlight how a reproducible and automated approach might be used to readily analyze global-scale metagenomic data sets and explore questions related to the functional potential and biogeographical distribution of eukaryotic marine communities.

### Eukaryotic genome recovery from Tara Oceans metagenomes spans major eukaryotic supergroups

**Fig 1:**
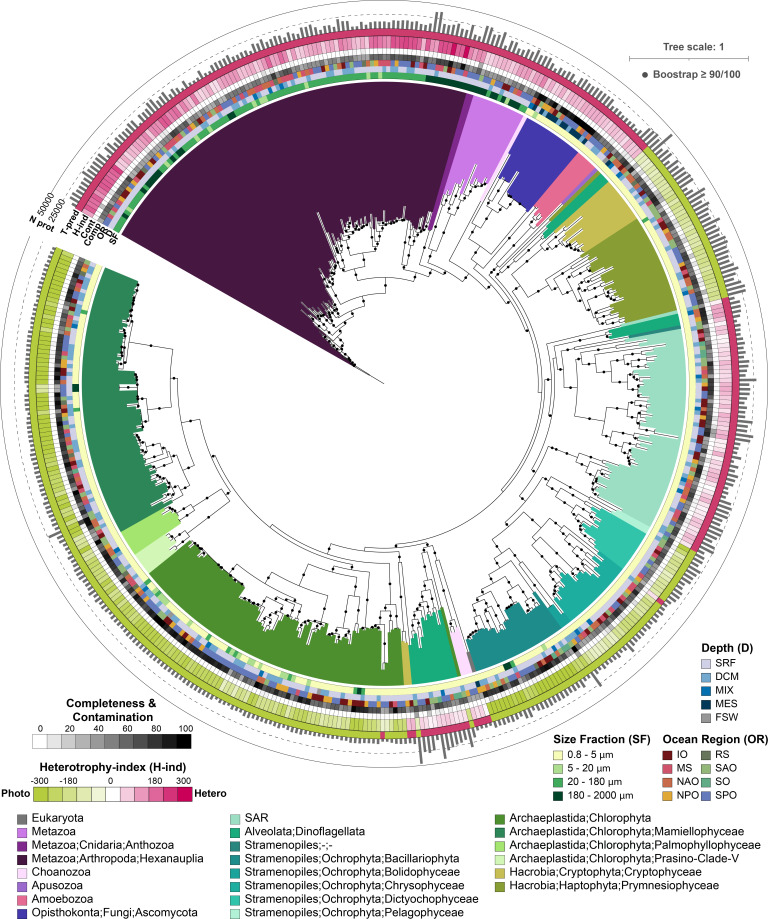
TOPAZ eukaryotic MAGs span the eukaryotic tree of life. The maximum likelihood tree was inferred from a concatenated protein alignment of 49 proteins from the eukaryotic BUSCO gene set (eukaryota_odb10) that were found to be commonly present across at least 75% of the 485 TOPAZ eukaryotic MAGs that were estimated to be 30% complete based on BUSCO ortholog presence (highly complete). The MAG names were omitted but the interactive version of the tree containing the MAG names can be accessed through iTOL (https://itol.embl.de/shared/halexand). Branches (nodes) are colored based on consensus protein annotation estimated by EUKulele and MM-Seqs. The OR, D, and SF of the co-assembly that a MAG was isolated from are color coded as colored bars. The completeness (comp), or percentage of the 255 eukaryotic BUSCOs present in a MAG, and contamination (cont), or over-representation (more than one copy) of eukaryotic BUSCOs in a MAG, are depicted as a heatmap. Predicted heterotrophy index (H-index), which ranges from phototroph-like (-300) to heterotroph-like (300) is shown as a heatmap. The predicted trophic mode (T-pred) based on the trophy random forest classifier with heterotroph (pink) and phototroph (green) is depicted. The number of proteins predicted with EukMetaSanity is shown as a bar graph along the outermost ring.

The EukHeist classification pipeline identified 988 putative eukaryotic MAGs following the refinement of recovered metagenomic bins based on length (>2.5 Mbp) and proportion of base pairs predicted to be eukaryotic in origin by EukRep (22) (Fig. S4). Protein coding regions in the eukaryotic MAGs were predicted using the EukMetaSanity pipeline (23), and the likely taxonomic assignment of each bin was made with MMSeqs (24) and EUKulele (25) (Table S2). Of the 988 eukaryotic MAGs recovered, 713 MAGs were estimated to be more than 10% complete based on the presence of core eukaryotic genes from the eukaryotic Benchmarking Universal Single-Copy Orthologs (BUSCO ) gene set(26). For the purposes of our subsequent analyses, we only consider the highly complete eukaryotic TOPAZ MAGs, or those that were greater than 30% complete based on BUSCO ortholog presence (*n* = 485) (Fig. 1).

**Fig 2:**
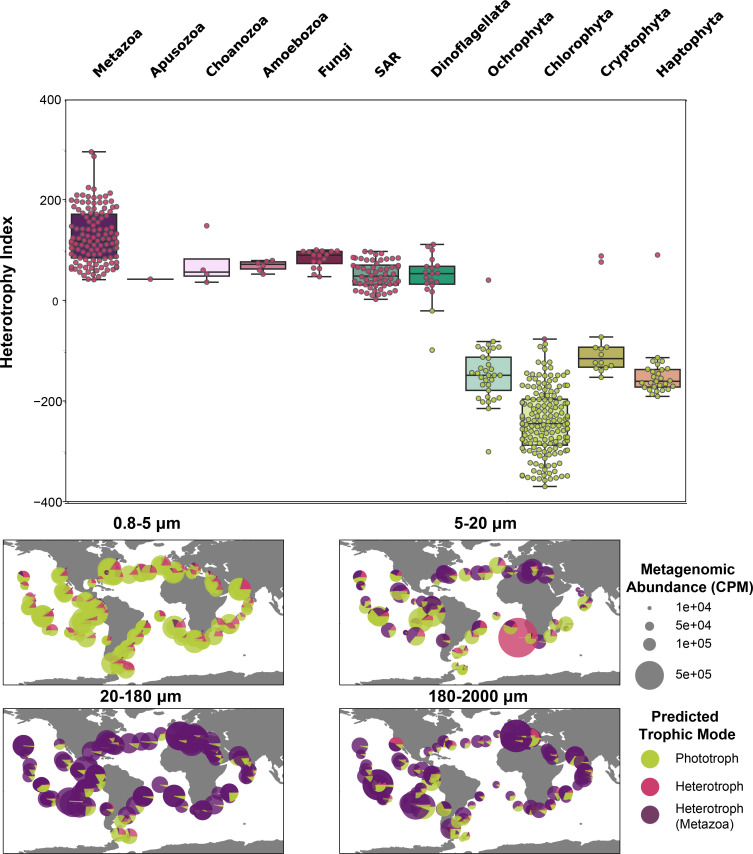
Estimated trophic status of TOPAZ eukaryotic MAGs. (Top) Trophic status was predicted for each high-completion TOPAZ eukaryotic MAG using a Random Forest model trained on the presence and absence of KEGG orthologs and is shown as a color (green, phototroph, pink, heterotroph). The heterotrophy index (H-index) (equation 8) for each MAG is plotted with a box plot showing the range of the H-index for each higher-level group. (Bottom) The relative distribution and abundance of phototroph (green), non-metazoan heterotroph (pink), and metazoan heterotroph (purple) are depicted across all surface samples. Plots are subdivided by size classes. “SAR” denotes MAGs with taxonomy assignments that were not resolved beyond the SAR group (Stramenopile, Alveolate, or Rhizaria).

Eukaryotic genomes are known to be both larger and have higher proportions of non-coding DNA than bacterial genomes (20). On average, across sequenced eukaryotic genomes, 33.1% of genomic content codes for genes (2.6%–59.8% for the first and third quartiles), while bacterial genomes have a higher proportion of coding regions (86.9%; 83.9%–89.3%) (27). The highly complete TOPAZ eukaryotic MAGs have an average of 73.7% ± 14.3% gene coding regions (Fig. S9). This trend of a higher proportion of coding regions was consistent across eukaryotic groups, where Haptophyta and Ochrophyta TOPAZ MAGs had an average coding region of 80.3% ± 4.9% and 78.1% ± 6.3%, respectively. Genomes from cultured Haptophyta (*Emiliania huxleyi* CCMP1516 with 31 Mb or 21.9% [28]) and Ochrophyta (*Phaeodactylum tricornutum* with 15.4 Mb or 57.3% [29]) had significantly lower proportions of protein-coding regions within their genomes compared to TOPAZ MAGs. The lowest percentages of gene coding were within Metazoan and Fungal TOPAZ MAGs, with 52.6% ± 9.8% and 58.8% ± 6.7%, respectively. As a point of comparison, the human genome is estimated to have ≈34 Mb or 1.2% of the genome coding for proteins (30). Globally, the higher gene coding percentages for the recovered eukaryotic TOPAZ MAGs likely reflect biases caused by the use of tetranucleotide frequencies in the initial binning (9) as well as challenges inherent in the assembly of non-coding and repeat-rich regions of eukaryotic genomes.

In order to evaluate the taxonomic breadth represented in the TOPAZ MAGs, estimated taxonomy of each MAG based on protein-consensus annotation was used for phylogenetic placement of TOPAZ MAGs (Fig. 1). The recovered MAGs spanned eight major eukaryotic supergroups: Archaeplastida (Chlorophyta), Opisthokonta (Metazoa, Choanoflagellata, and Fungi), Amoebozoa, Apusozoa, Haptista (Haptophyta), Cryptista (Cryptophyta), and lineages collectively referred to as the SAR supergroup (Stramenopiles, Alveolata, and Rhizaria) (31), similar to other eukaryotic MAG recovery efforts from the Tara Oceans data set (Fig. S14) (17). Eukaryotic MAGs were retrieved from all ocean regions surveyed, with the largest number of highly complete TOPAZ MAGs recovered from the South Pacific Ocean Region (*n* = 143) and the fewest recovered from the Southern Ocean (SO) (*n* = 11) and Red Sea (RS) (*n* = 12) (Fig. S8). These regional trends in MAG recovery and taxonomy aligned with the overall sequencing depth at each of these locations (Table S1), with fewer, less diverse MAGs recovered from the SO and RS (Fig. S5 and S8).

The largest number of highly complete MAGs was recovered from the smallest size fraction (0.8–5 µm) (*n* = 311) (Fig. 1; Fig. S5) and yielded the highest taxonomic diversity, including MAGs from all the major supergroups listed above (Fig. S5). The groups that made up the largest proportion of small-size fraction MAGs were Chlorophyta (*n* = 133), Ochrophyta (*n* = 33), or taxa associated with the SAR group (Stramenopiles, Alveolata, and Rhizaria; *n* = 56). Chlorophyta MAGs were smaller and had fewer predicted proteins relative to other eukaryotic MAGs, despite demonstrating comparable completeness metrics; the average Chlorophyta MAG size was 13.9 Mbp with 7,525 predicted proteins (Fig. S6 and S9). By contrast, Cryptophyta and Haptophyta had the largest average MAG size with 50.8 and 44.4 Mbp with an average of 23,500 and 24,400 predicted proteins, respectively (Fig. S9). Fewer eukaryotic MAGs were recovered from the other size fractions 5–20 µm (*n* = 20), 20–180 µm (*n* = 87), and 180–2,000 µm (*n* = 39) (Fig. S5); instead, these larger size fractions recovered a higher total number of metazoan MAGs. Metazoan MAGs had the lowest average completeness (50% ± 13%) (Fig. S7 and S9), where the average size of recovered metazoan MAGs was 43.2 Mb (6.5–177 Mbp), encompassing an average of 14,600 proteins (Fig. S9). Of the 123 metazoan MAGs, 76 likely belong to the Hexanauplia (Copepoda) class; copepod genomes have been estimated to be up to 2.5 Gb with high variation (10-fold difference) across sequenced members (32). The taxonomic composition of EukHeist-recovered TOPAZ MAGs aligned with what might be expected based on the size fraction and depth from which they were isolated.

MAGs were also retrieved from all discrete sampling depths: surface, SRF (*n* = 315), deep chlorophyll max, DCM (*n* = 133), mesopelagic, MES (*n* = 13), as well as samples with no discrete depth, MIX (*n* = 21) and the filtered seawater controls, FSW (*n* = 3). Notably, the filtered seawater included one Chlorophyta MAG (TOPAZ_IOF1_E003), which was estimated to be 100% complete with no contamination (Table S2). These results suggest that variables such as *in situ* diversity, cell size (and genomic size), and sampling protocols influence our ability to obtain high-quality and highly complete eukaryotic MAGs.

The composition of TOPAZ MAGs from basin-scale mesopelagic co-assemblies recovered a higher percentage of fungi relative to other depths. This is similar to other mesopelagic and bathypelagic molecular surveys, where the biomass of fungi is thought to outweigh other eukaryotes (33–35). Furthermore, fungal MAGs had the highest overall average completeness (87% ± 15%) (Fig. S7 and S9). A total of 16 highly complete fungal MAGs were also recovered; of those, 11 originated from the mesopelagic (Fig. 1; Fig. S8). Putative fungal TOPAZ MAGs were recovered from the phyla Ascomycota (*n* = 10) and Basidiomycota (*n* = 1) and ranged in size from 12.5 to 47.8 Mb (Fig. S9), which are within the range of known average genome sizes for these groups, 36.9 and 46.5 Mb, respectively (36).

The metagenomic read recruitment to the eukaryotic TOPAZ MAGs paralleled MAG recovery, where metazoan MAGs dominated the larger size fractions (20–180 µm and 180–2,000 µm) across both the surface and DCM for all stations, and Chlorophyta MAGs were dominant across most of the small size fraction stations (0.8–5 µm) (Fig. S11). A notable exception were the stations from the Southern Ocean, where Haptophyta and Ochrophyta were the most abundant taxa across all size fractions. Given the proponderance of bloom-forming *Phaeocystsis antarctica* and chain-forming diatom taxa (37), the broad distribution of MAGs across all size fractions is unsurprising. With respect to depth, the average recruitment of reads from the mesopelagic was far lower than the photic zone (surface and DCM); the average CPM across mesopelagic samples was 24,500 ± 34,450, while surface and DCM samples recruited 131,000 ± 104,00 and 136,00 ± 85,000 reads, respectively (Fig. S11). The low read recruitment demonstrates how binning MAGs may not capture the entire eukaryotic community; in the case of the mesopelagic, this can be explained by the presence of highly diverse and distinct microbial populations (34), or that the mesopelagic communities sampled were dominated by prokaryotic biomass (38).

### Eukaryotic MAG gene content can be used to predict trophic status

Eukaryotic microbes can exhibit a diversity of functional traits and trophic strategies in the marine environment (1, 39, 40), including phototrophy, heterotrophy, and mixotrophy. Phototrophic protists are responsible for a significant fraction of organic carbon synthesis via primary production; these phototrophs dominate the microbial biomass and diversity in the sunlit layer of the oceans (39, 41). Phagotrophic protists (heterotrophs), which ingest bacteria, archaea, and smaller eukaryotes, and parasitic protists are known to account for a large percentage of mortality in food webs (1, 39, 42). Protists are also capable of mixed nutrition (mixotrophy), where a single cell exhibits a combination of phototrophy and heterotrophy (43). Typically, the identification of trophic mode has relied upon direct observations of isolates within a lab setting, with more recent efforts including transcriptional profiling as a means of assessing trophic strategy (44, 45). Scaling up these culture-based observations to environmentally relevant settings (46–49) has been an important advance in the field for exploring complex communities without cultivation. An outcome of these studies has been the realization that trophic strategies are not governed by single genes (50); in reality, trophic strategy will be shaped by an organism’s physiological potential and environmental setting. Therefore, larger genomic and transcriptomic efforts to predict or characterize presumed trophic strategies among mixed microbial communities will greatly contribute to our understanding of the role that microorganisms play in global biogeochemical cycles, by enabling the observation of functional traits and strategies *in situ*.

Large-scale meta’omic results, such as the TOPAZ MAGs recovered here, can be leveraged alongside presently available reference data to enable the prediction of biological traits (such as trophic mode) without *a priori* information. Machine learning (ML) applications can be implemented to access the potential of these large data sets. ML approaches have been recently shown to be capable of accurate functional prediction and cell type annotation using genetic input, in particular, for cancer cell prediction (51–53), and functional gene and phenotype prediction in plants (54). Recently, these approaches have been applied to culture and environmental transcriptomic data to predict trophic mode using currently available trophy annotations (55–58). Here, we apply an independent machine learning model to the eukaryotic TOPAZ MAGs to predict each organism’s capacity for various metabolisms.

We used a variable selection algorithm and Random Forest machine learning model framework to predict the likely trophic mode of the eukaryotic TOPAZ MAGs described in this study. Transcriptomes from the MMETSP and EukProt were manually annotated as phototroph, mixotroph, or heterotroph based on the literature (Data set S1 at https://osf.io/twz2f/). We tested our model with a randomly selected test set comprising 25% of MMETSP and EukProt transcriptomes (44, 59) that were excluded from the model-building procedure. With this test subset, we obtained an accuracy of 94.6% (Fig. S16), meaning that nearly 95% of taxonomic annotations derived from the machine learning model aligned with their manually assigned trophic mode annotation (Fig. S16). Thus, for all sufficiently complete (≥30%) TOPAZ MAGs, we have predicted a gross trophic category (heterotrophic [*n* = 227], mixotrophic [*n* = 0], or phototrophic [*n* = 258]). Notably, all MAGs were either classified as phototrophs or heterotrophs, with none classified as mixotrophs. This likely reflects that the model was generally conservative when it came to assigning genomes or transcriptomes as mixotrophs (Fig. S20). Broadly, the trophic predictions aligned well with the putative taxonomy of each MAG (Fig. 1 and 2). For example, TOPAZ MAGs that had taxonomic annotation of well-known heterotrophic lineages (Metazoa, Fungi) were predicted as heterotrophs by our model. Moreover, our data-driven trophic mode predictions correlate well with an independent model designed to identify the presence of photosynthetic machinery and capacity for phagotrophy (55, 56) (Fig. S19 and S20).

As the gradient of trophic mode among protists is not strictly ternary (heterotrophic, autotrophic, and mixotrophic) and continues to be refined (40), we also calculated a heterotrophy index (H-index) that places the TOPAZ MAGs on a scale of highly phototrophic (negative values) to highly heterotrophic (positive values) (Fig. 1 and 2). The H-index assesses the extent of heterotrophy in the test genomes and transcriptomes using Kyoto Encyclopedia of Genes and Genomes (KEGG) Orthologs (KOs) selected by the feature selection process (*n* = 1,787). Instead of using the presence or absence of these KOs as a binary indicator to inform the classification of the MAGs (as above), we included their presence or absence in an equation to more sensitively assess the number of KOs present, which tended to be indicative of either heterotrophy or phototrophy. The resulting H-index is a metric for assessing trophy based on KEGG pathway presence or absence. Despite evidence that many lineages recovered include known mixotrophs, no TOPAZ MAGs were identified as mixotrophic using this approach. The H-index allows us to identify potential mixotrophy-capable MAGs via a descriptive scale rather than a ternary classification. We explore the likely reasons for this more deeply in Section 2.3 of the supplemental material, but one potential explanation is that MAG recovery targets the genome content of a eukaryotic lineage. The evolutionary history of phototrophy and heterotrophy is complicated and varies by species (60). Therefore, the genetic composition of MAGs may reflect encoded metabolisms that are not necessarily exhibited *in situ*. Mixotrophy is not a singular trait, but rather a spectrum of metabolic abilities that are largely driven by the microorganism’s nutritional needs and surrounding environment.

This work demonstrates the value of large untargeted genetic approaches to gain insight into the *in situ* metabolisms of less explored branches of the eukaryotic tree of life. Automated recovery of eukaryotic MAGs, independent of a reference database, and the trophic mode prediction demonstrate how we can begin to parse the metabolic contributions of individual eukaryotes to mixed microbial communities. While we cannot confidently annotate beyond specific taxonomic levels or protein identities, our ML model approaches still allow us to capture predicted nutritional strategies alongside the environmental context provided by the large-scale global sampling effort. Continued culturing efforts combined with large-scale meta’omic studies will continue to improve such ML models focused on complex traits and ultimately our ability to predict trophic mode. We suggest that the integration of metagenomic and metatranscriptomic data sets might better reflect the active strategies being used.

### TOPAZ prokaryotic MAGs distinct from previous marine MAG recovery efforts

**Fig 3:**
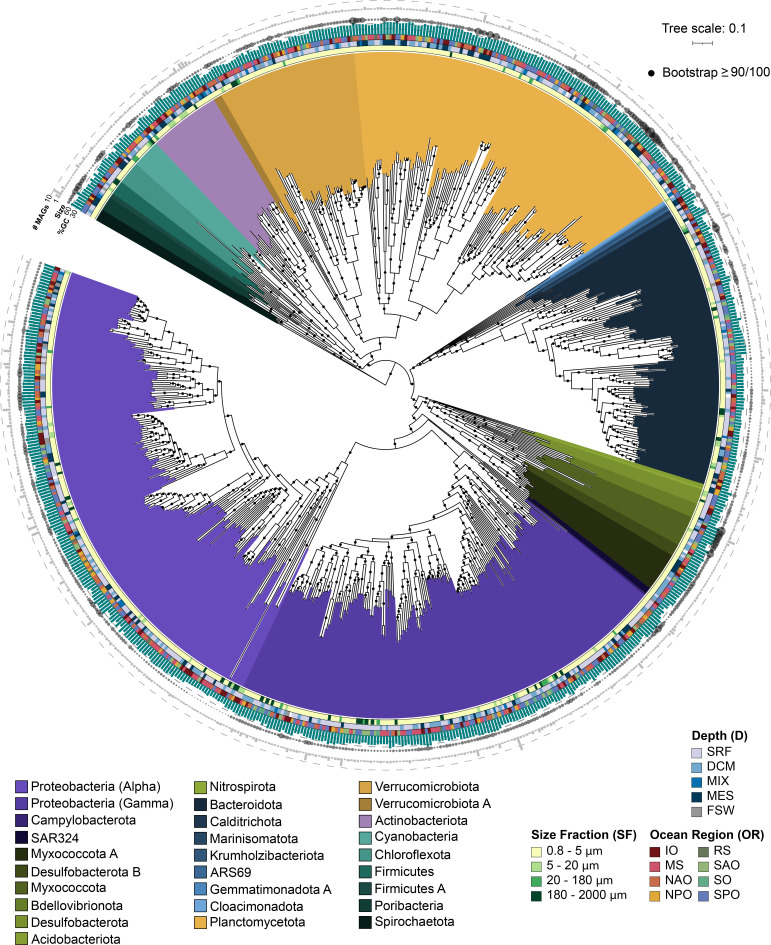
Diversity of the high-quality non-redundant bacterial TOPAZ MAGs. The approximately maximum-likelihood phylogenetic tree was inferred from a concatenated protein alignment of 75 proteins using FastTree and GToTree workflow. The MAG names were omitted but the interactive version of the tree containing the MAG names can be accessed through iTOL (https://itol.embl.de/shared/halexand). Branches (nodes) are colored based on taxonomic annotations estimated by GTDBtk. The OR, SF, and D of the co-assembly that a MAG was isolated from is color coded as colored bars. The GC (%) content is shown as a bar graph (in green), the genome size as a bubble plot (the estimated size of the smallest genome included in this tree is 1.00 Mbp and the largest is 13.24 Mbp), and the number of MAGs in each genomic cluster (of 99 or higher %ANI) as a bar plot (in gray).

Preliminary bins from EukHeist totaled over 16,000, of which a large percentage were prokaryotic MAGs, specifically Bacteria. A major utility of EukHeist is that results will include bins from all three domains of life. While EukHeist will work to filter putative eukaryotic MAGs, it will also process and analyze bacteria and archaeal bins. High-quality non-redundant TOPAZ (HQ-NR-TOPAZ) MAGs comprises 711 bacterial and 5 archaeal MAGs belonging to 30 different phyla (Fig. 3; Table S4); an additional 15 phyla were recovered in the medium-quality (MQ) MAGs. Of the 716 HQ-NR-TOPAZ MAGs, 507 were unique based on a 99% average nucleotide identity (ANI) comparison threshold with MAGs generated from previous binning efforts from Tara Oceans metagenomic data, including Delmont et al. (12) (TARA), Tully et al. (13) (TOBG), and Parks et al. (11) (UBA) (Fig. 3). The phylogenetic diversity captured by the TOPAZ MAGs was quantified by comparison to a “neutral” reference set of genomes; these neutral references approximate the state of marine microbial genomes, dominated by isolate genomes, previous to the incorporation of the Tara Oceans-derived MAGs (Table 1). Relative to the neutral genomic references, the entire TOPAZ NR (includes both HQ and MQ) set represented a 42.8% phylogenetic gain (as measured by additional branch length contributed by a set of data) and 59.9% phylogenetic diversity (as measured by the total branch length spanned by a set of taxa), as compared to efforts focused solely on the smaller size fractions such as TARA and UBA, which had a smaller degree of gain (31.0% and 25.8%, respectively) and diversity (44.4% and 40.5%, respectively) (Table 1). An inclusive tree containing the neutral reference and all Tara Oceans MAGs (TOBG + UBA + TARA + TOPAZ), the TOPAZ NR MAGs represented 14.4% of the phylogenetic gain and 44.7% phylogenetic diversity, suggesting that the TOPAZ MAGs offer the largest increase in phylogenetic novelty when compared to MAGs reconstructed from the metagenomes of the smaller size fractions (<5.00 µm). The TOPAZ MAGs primarily originated from the larger Tara Ocean size fraction samples, and thus include a higher proportion of more complex host- and particle-associated bacterial communities. The novelty of the HQ- and MQ-NR-TOPAZ MAGs here suggests that these particle-associated MAGs are overlooked, and current genome databases are largely skewed toward free-living bacteria.

**TABLE 1:** Phylogenetic diversity and gain of various MAGs originating from Tara Oceans^*d*^

Base tree^*c*^	MAGs of interest	No. of MAGs	Phylogenetic diversity^*a*^ (%)	Phylogenetic gain^*b*^ (%)
Neutral	TOPAZ (MQ, NR)	1,571	59.9	42.8
Neutral	TOPAZ (HQ, NR)	634	41.6	25.8
Neutral	TOBG	1,974	61.3	46.7
Neutral	UBA	1,052	40.5	25.8
Neutral	TARA	722	44.4	31.0
Neutral	TOBG +UBA + TARA	3,750	66.6	51.8
Neutral + Tara Oceans MAGs,HQ	TOPAZ (HQ, NR)	634	26.1	6.2
Neutral + Tara Oceans MAGs, MQ	TOPAZ (MQ, NR)	1,572	44.7	14.4
Neutral + Tara Oceans MAGs, MQ	TOBG	1,977	48.5	11.1
Neutral + Tara Oceans MAGs, MQ	UBA	1,055	23.8	1.6
Neutral + Tara Oceans MAGs, MQ	TARA	722	28.0	3.4

^*a*^ Total branch length spanned by a set of taxa.

^*b*^ Additional branch length contributed by a set of taxa.

^*c*^ HQ in base tree indicates that it includes Neutral, TOBG, UBA, TARA, TOPAZ HQ, and NR MAGs, and MQ in base tree indicates that it includes Neutral, TOBG, UBA, TARA, TOPAZ MQ, and NR MAGs.

^*d*^ Phylogenetic diversity and gain of prokaryotic MAGs were assessed for this study (TOPAZ), TOBG (13), UBA (11), and TARA (12) relative to each other as well as a “Neutral” tree comprising relevant marine bacteria.

To confirm the hypothesis that the prokaryotic TOPAZ MAGs included particle-associated members, we examined the genomic features of several selected groups that were well-recovered here and in single-cell amplified genomic (SAG) data sets (i.e., Global Ocean Reference Genomes [GORG]) (61). To avoid potential biases related to completeness and contamination of the genomes, only the HQ-NR MAGs were compared to the GORG SAGs, and analyses were limited to groups with sufficient representation within both data sets (Bacteriodota, Cyanobacteria, and Proteobacteria). For these well-represented groups, the average GC% and estimated genome size of the TOPAZ MAGs were significantly higher than the ones typically reported in free-living marine bacteria (62–64) and those observed within the GORG data set (61). TOPAZ MAGs were found to encode more tRNAs on average per genome than GORG (39.5 vs 30). Additionally, carbohydrate-active enzymes and peptidases were enriched within the TOPAZ MAGs relative to GORG (Fig. S22). Larger genomes have been considered diagnostic for a copiotrophic lifestyle in bacteria (65), since the more extended and flexible gene repertoire can facilitate substrate catabolism in organic-rich niches such as particles. Genomes of copiotrophs are also commonly found to have higher copy numbers of genes associated with replication and protein biosynthesis such as tRNAs and rRNAs (66), which facilitate higher growth rates. In contrast, the streamlined genomes of SAR11 and other groups that have free-living oligotrophic lifestyles require fewer resources to maintain and replicate their genomes and have higher carbon-use efficiency (67). Similarly, G and C have higher energy cost of production and more limited intracellular availability compared to A and T (64, 68). The genomic trends observed support our findings that TOPAZ MAGs represent both particle-associated and free-living microbes and are relatively enriched for copiotrophic microbes.

### Environmental factors structure TOPAZ MAG co-occurrence

**Fig 4:**
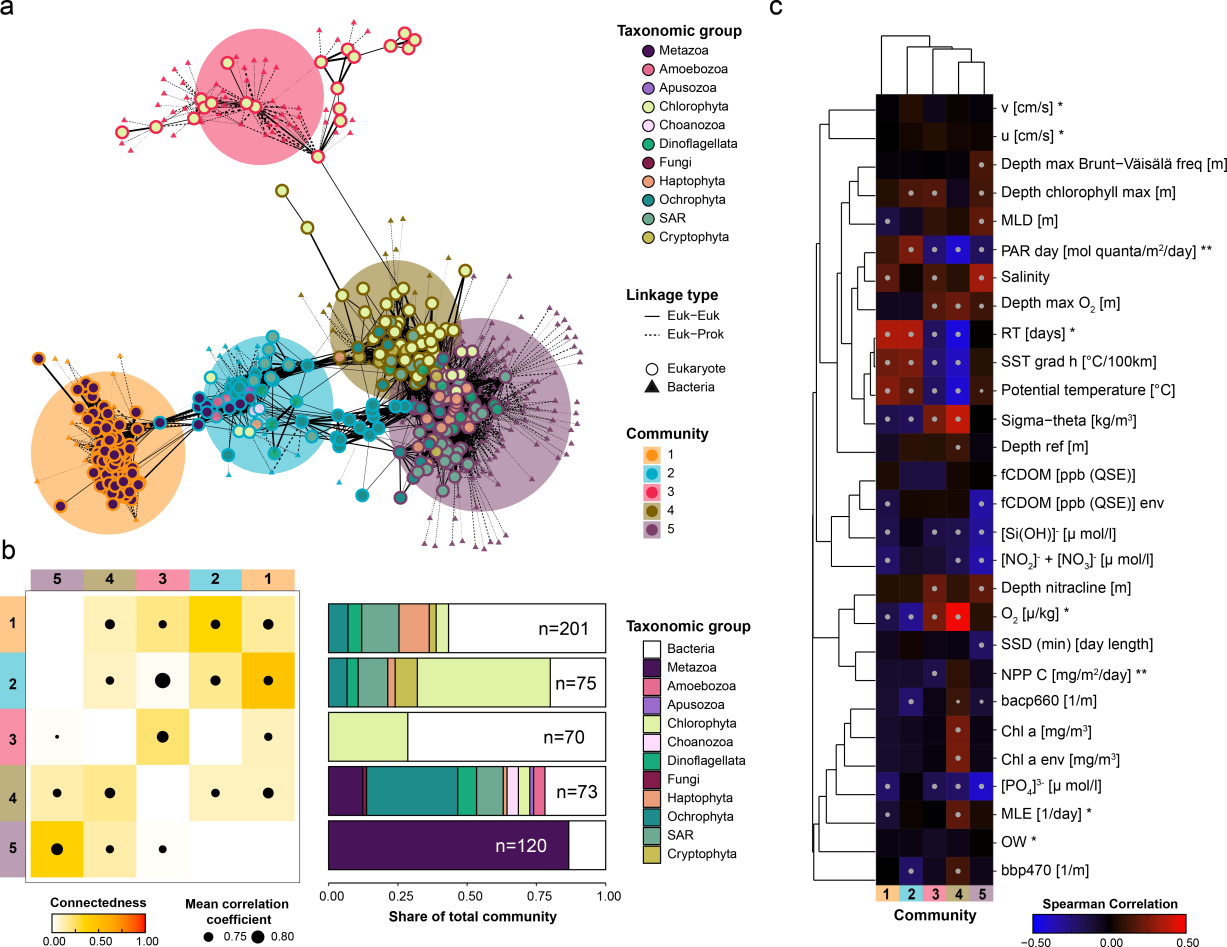
Distinct communities recovered from the TOPAZ MAGs. (a) A network analysis performed on the metagenomic abundance of all recovered eukaryotic and prokaryotic TOPAZ MAGs based on Spearman correlation analysis, identifying five distinct communities (see Materials and Methods). A force-directed layout of the seven communities is shown with eukaryotes (circles) and bacteria (triangles). Only linkages between eukaryotes are visualized. (b) The connectedness and taxonomic composition of each community are depicted. Connectedness was calculated based on equations 9)–11. (c) A Spearman correlation between the summed metagenomic abundance of each community and environmental parameters from the sampling (70), modeled mesoscale physical features based on d’Ovidio et al. (71) (indicated with *), and averaged remote sensing products (indicated with **). Significant Spearman correlations, those with a Bonferroni adjusted *P* < 0.01, are indicated with a dot on the heatmap.

The co-retrieval of eukaryotic and prokaryotic MAGs from across the global ocean allows the unique opportunity to assess the biogeographical and ecological associations and potential co-occurrence of these organisms while also being able to infer likely functions. To identify communities of associated organisms that co-occur across the surface ocean metagenomes, we performed a correlation clustering based on the abundances of the eukaryotic TOPAZ MAGS and the HQ-NR-TOPAZ MAGs (Fig. 4a). We employed a modularity optimization algorithm to the correlation analysis (69) to identify distinct communities of co-occurring organisms. This approach identified five distinct communities (Fig. 4b). The communities were variably connected to each other, as defined by equations 9–11, with the highest connectedness between communities 1 and 2, and 4 and 5 (Fig. 4b; the maximum connectedness between 1 and 2 was 0.233, and the maximum connectedness between 4 and 5 was 0.448). Community 3 showed the lowest degree of connectivity within community members and to other communities (mean = 0.108; remaining community mean = 0.248), suggesting that members of this community co-occur less consistently across samples.

The five communities that we identified based on metagenomic abundance correlations also significantly correlated with environmental factors, which consequently define the environmental niches where the communities were most abundant (Fig. 4c; Table S9). Temperature (Tpot, which ranged from −1.6 to 30°C) was a primary factor defining the community correlations, significantly correlating with four of the five communities. Communities 2 and 4 correlated with colder temperatures (Fig. 4c). For Community 4, there was a significant positive correlation with chlorophyll (Chla: ρ = 0.236, *P* = 3.69*e*−11), while we found negative correlations with “residence time” (ρ = −0.438, *P* = 1.61*e*−24), indicating a likely occurrence in newly formed eddies (according to the calculation by d’Ovidio et al. [71] as reported in the Tara Oceans metadata [(70] as “residence time”). This aligns with the finding that Community 4 was typically found within colder, productive regions and had higher metagenomic abundances in the Southern Ocean and the North Atlantic (Fig. S24). The composition of Community 4 MAGs included Chlorophyta, Cryptophyta, Haptophyta, and Ochrophyta, the major groups containing primarily phototrophic eukaryotic microbes. A total of 19 prokaryotic MAGs were also contained in this community, including both photosynthetic (Synechococcales) and non-photosynthetic lineages (e.g., Myxococcota and Planctomycetota). This guild of MAGs comprises likely photosynthesizers often found in cold but not necessarily nutrient-rich, environments. Communities 1 and 3 correlated with warmer temperatures (Fig. 4c), which was attributed to their presence in longer-lived eddies (Fig. 4c; community 1: ρ = 0.349, *P* = 1.19*e−*14; community 3: ρ = 0.345, *P* = 2.51*e−*14). However, these two communities differed both in their association with nutrients and their taxonomic compositions. Community 1 was dominated by Metazoa and bacteria and correlated with low nutrient (oligotrophic) conditions (nitrate and nitrite:ρ = −0.218, *P* = 3.36*e*−9; phosphate: ρ = −0.218. *P* = 3.39*e−*9 and silica: ρ = −0.157, *P* = 1.96*e*−4) and was most abundant in the larger size fraction samples (20–2,000 µm) (Fig. S24). In contrast, community 3 largely comprised phototrophic chlorophytes and bacteria and was not significantly correlated with nutrient conditions and was most abundant in smaller size fraction samples (0.8–20 µm), particularly around the tropics (Fig. S24). Community 5 was weakly associated with warmer water (ρ = 0.108, *P* = 9.109*e−*2) and comprised SAR and bacteria (Fig. 4b). Additionally, community 5 was negatively correlated with nutrient concentrations (nitrate and nitrite: ρ = −0.348, *P* = 2.14*e*−25; phosphate: ρ = −0.407, *P* = 7.36*e*−36; and silica: ρ =−0.310, *P* = 7.62*e*−20), suggesting that this community thrives in oligotrophic regions.

While many of the communities recovered appeared to be driven largely by environmental forces, the taxonomic affiliation of community 1 members suggests that this community comprised MAGs indicative of a eukaryotic host with an associated bacterial microbiome. Community 1 comprised primarily of metazoan, specifically Hexanauplia, and bacterial MAGs (Fig. 4b). Many of the bacterial MAGs in community 1 had genes that suggest adaptations to microaerophic niches such as those which might be experienced when living in close host association (e.g., high-affinity oxygen cytochromes and reductases) (Fig. S26). The bacterial MAGs in community 1 could be broadly broken into two apparent functional types: those with larger genomes typical of copiotrophic bacteria and those with small genomes indicative potentially of reductive evolution. The first group comprised MAGs from the family Saprospiraceae in phylum Bacteriodota (*n* = 2, 3.0 Mbp average genome size), the order Opitutales in phylum Verrucomicrobiota (*n* = 2, 3.4 Mbp), and the family Vibrionaceae (*n* = 2, 4.5 Mbp) in phylum Proteobacteria (Fig. S26). In addition to their relatively large size, the Saprospiraceae and Vibrionaceae MAGs were found to encode for genes involved in the hydrolysis and utilization of various complex carbon sources including chitin and other carbohydrates (Fig. S26), such as those that might be shed or excreted by zooplankton such as copepods (72). By contrast, the second group of bacterial MAGs within community 1 with smaller genomes included MAGs from the Proteobacteria order Rickettsiales (*n* = 3, 0.6–1.2 Mbp) and the Gammaproteobacteria family Francisellaceae (*n* = 1, 1.2 Mbp) (Fig. S26). The smaller genome sizes exhibited by these groups may be indicative of a genome streamlining, which occurred with reductive evolution due to obligate or facultative symbiosis (67). Rickettsiales and Francisellaceae contain well-described obligate intracellular symbionts (73–75) and zoonotic pathogens (74, 76).

### Conclusion

Sequence data sets are revolutionizing how we form new hypotheses and explore environments on the planet. Here, we demonstrated a critical advance in the recovery of MAGs from environmentally relevant eukaryotic organisms with EukHeist. The retrieval of MAGs to investigate the role of microorganisms in biogeochemical cycles in natural environments is promising; however, the current lack of eukaryotic reference genomes and transcriptomes complicates our ability to interpret the eukaryotic component of the microbial community. We recovered 988 total eukaryotic MAGs, 485 of which were deemed highly complete, and over 4,000 prokaryotic MAGs, which had signatures of particle association. Our findings demonstrate that specific branches of the eukaryotic tree were more likely to be resolved at the MAG level due to their smaller genome size, distribution in the water column, and biological complexity. A substantial portion of the recovered eukaryotic MAGs were distinct from existing sequenced representatives, demonstrating that these large-scale surveys are a critical step toward characterizing less-resolved branches of the eukaryotic tree of life.

The continuing expansion of global-scale meta’omic surveys, such as BioGeoTraces (77), Bio-GO-SHIP (78, 79), and the continuation of the Tara Oceans program (80–82), highlights the importance of developing scalable and automated methods to enable a more complete analysis of these data. Metagenomic pipelines that specifically integrate steps for handling eukaryotic biology, such as the EukHeist pipeline, are vital as eukaryotes are important members of microbial communities, ranging from the ocean to soil (83) and human- (84) and animal-associated (85) environments. Additionally, we aim to contribute computational tools that can be integrated or customized, including EUKulele, EukMetaSanity, and eukrhythmic (23, 25, 86). The application of eukaryotic-sensitive methods such as EukHeist to other systems stands to greatly increase our understanding of the diversity and function of the “eukaryome.”

## MATERIALS AND METHODS

### Data acquisition

The metagenomic and metatranscriptomic data corresponding to the size fractions dominated by eukaryotic organisms ranging from microbial eukaryotes and zooplankton (0.8–2,000 µm) as originally published by Carradec et al. (21) were retrieved from the European Molecular Biology Laboratory-European Bioinformatics Institute (EMBL-EBI) under the accession numbers PRJEB4352 (large size fraction metagenomic data) and PRJEB6603 (large size fraction metatranscriptomic data) on 20 November 2018. Only samples with paired-end reads (forward and reverse) were used in the subsequent analyses (Table S1). After an initial sample-to-sample comparison with sourmash (sourmash compare -k 31 -scaled 10,000) (87) (Fig. S3), it was determined that samples largely clustered by depth and size fraction. Samples were grouped for co-assembly by size fraction (0.8–5 µm, 5–20 µm, 20–180 µm, and180–2,000 µm) as per reference 21 depth or sample type (SRF, DCM, MES, MIX, and FSW) and geographic location (Table S1). In cases where a sample did not fall directly within one of the size classes, it was assigned to an existing size class based on the upper micrometer limit of the sample. This grouping resulted in the combination of 824 cleaned, paired FASTQ file samples into 94 distinct co-assembly groups, which were used downstream for co-assembly (Table S1).

### EukHeist pipeline for metagenome assembly and binning

The metagenomic analysis, assembly, binning, and all associated quality control steps were carried out with a bioinformatic pipeline, EukHeist, which enables user-guided analysis of stand-alone metagenomic or paired metagenomic and metatranscriptomic sequence data. EukHeist is a streamlined and scalable pipeline currently based on the Snakemake workflow engine (88) that is configured to facilitate deployment on local HPC systems. Figure S2 outlines the structure and outputs of the existing EukHeist pipeline. EukHeist is designed to retrieve and identify both eukaryotic and prokaryotic MAGs from large, metagenomic and metatranscriptomic data sets (Fig. S2). EukHeist takes input of sequence meta-data, user-specified assembly pairings (co-assembly groups), and raw sequence files and returns MAGs that are characterized as either likely eukaryotic or prokaryotic.

Here, all raw sequences accessed from the EMBL-EBI were quality assessed with FastQC and MultiQC (89). Sequences were trimmed using Trimmomatic (v.0.36; parameters: ILLUMINACLIP: 2:30:7, LEADING:2, TRAILING:2, SLIDINGWINDOW:4:2, MINLEN:50) (90). Passing mate paired reads were maintained for assembly and downstream analyses. Quality trimmed reads co-assembled based on assembly groups (Table S1) with MEGAHIT (v1.1.3, parameters: *k* = 29, 39, 59, 79, 99, 119) (91). Basic assembly statistics were assessed for all co-assemblies with Quast (v. 5.0.2) (92) (Table S1). Cleaned reads from assembly-group-associated metagenomic and metatranscriptomic samples were mapped back against the assemblies with bwa mem (v.0.7.17) (93). The bwa-derived abundances were summarized with MetaBat2 (v. 2.12.1) script jgi_summarize_bam_contig_depths (with default parameters). The output contig abundance tables were used along with tetranucleotide frequencies to associate contigs into putative genomic bins using MetaBat2 (v. 2.12.1) (9). The Snakemake profile used to conduct this analysis is available at https://www.github.com/alexanderlabwhoi/tara-euk-metag. A generalized version of the Snakemake pipeline (called EukHeist) that might be readily applied to other data sets is available at https://www.github.com/alexanderlabwhoi/EukHeist. MAGs here are subsequently named and referred to as TOPAZ and are individually named based on their assembly group (Tables S2 and S3).

### Identification of putative eukaryotic MAGs

The binning process described above recovered a total of 16,385 putative bins. These bins were screened to identify high-completion eukaryotic and prokaryotic bins. All bins were first screened for length, assuming that eukaryotic bins would likely be greater than 2.5 Mbp in size [modeled off of the size of the smallest known eukaryotic genome, ∼2.3 Mbp *Microsporidian Encephalitozoon intestinalis* (94)]. Bins larger than 2.5 Mbp were screened for relative eukaryotic content using EukRep (22), a k-mer-based strategy that estimates the likely domain origin of metagenomic contigs. EukRep was used to classify the relative proportion of eukaryotic and prokaryotic content in each bin in a contig-by-contig manner. This approach identified 907 candidate eukaryotic bins that were greater than 2.5 Mb in length and estimated to have more than 90% eukaryotic content by length. Protein coding domains were predicted in all 907 putative eukaryotic bins using EukMetaSanity (23).

### Protein prediction in eukaryotic MAGs with EukMetaSanity

#### Taxonomy

The MMseqs2 v12.113e3 (24, 95, 96) taxonomy module (parameters: -s 7 --min-seq-id 0.40 -c 0.3 --cov-mode 0) was used to provide a first-pass taxonomic assignment of the input MAG for use in a downstream element of EukMetaSanity pipeline that requires an input NCBI taxon id or a taxonomic level (i.e., Order, Family, etc.). We created a custom database comprising both OrthoDB (97) and MMETSP (44) protein databases (OrthoDB-MMETSP) that integrate NCBI taxon ids. MMseqs2 was used to query each MAG against the OrthoDB-MMETSP database to identify a first-pass taxonomic assignment. The lowest common ancestor of top-scoring hits was identified to provide taxonomic assignment to each candidate eukaryotic bin. The taxonomyreport module generates a taxon tree that includes the percentage of MMseqs mappings that correspond to each taxonomic level. A taxonomic identifier and scientific name are selected to the strain level or when total mapping exceeds 8%, whichever comes first. The assigned NCBI taxon id is retained for downstream analyses.

#### Repeats identification

RepeatModeler (98, 99) was used to provide *ab initio* prediction of transposable elements, including short and long interspersed nuclear repeats, as well as other DNA transposons, small RNA, and satellite repeats. RepeatMasker (100) was then used to hard-mask these identified regions, as well as any Family-level (as identified above) repeats from the DFam 3.2 database (101). RepeatMasker commands ProcessRepeats (parameter: -nolow) and rmOutToGff3 (parameter: -nolow) were used to output masked sequences (excluding low-complexity repeat DNA from the mask) as FASTA and gene-finding format (GFF3) files, respectively.

#### *Ab initio* prediction

GeneMark (102) was used to generate *ab initio* gene predictions with the repeat-masked eukaryotic candidate bin sequences’ output from the prior step. The GeneMark subprogram ProtHint attempts to use Order-level proteins from OrthoDB-MMETSP database to generate intron splice-site predictions for *ab initio* modeling using GeneMark EP (103). If ProtHint fails to generate predictions, then GeneMark will default to ES mode. Due to the fragmented nature of metagenomic assemblies, the prediction parameter stringency was drastically reduced relative to what is recommended for draft genome projects (parameters: -min_contig 500 -min_contig_in_predict 500 -min_gene_in_predict 100). These parameters can be easily modified within the EukMetaSanity config file. GeneMark outputs predictions of protein-coding sequences (CDS) and exon/intron structure as GFF3 files.

#### Integrating protein evidence

MetaEuk (104) was used to directly map the repeat-masked eukaryotic candidate bin sequences against proteins from the MMETSP (44, 105) and eukaryotes included in the OrthoDB v10 data set (97), hereafter referred to as the OrthoDB-MMETSP database. MetaEuk easy-predict (parameters: -min-length 30 -metaeuk-eval 0.0001 -s 7 -cov-mode 0 -c 0.3 -e 100 -max-overlap 0) used Order-level proteins to identify putative CDS and exon/intron structure. MetaEuk encodes this output as headers in FASTA sequences that are then parsed into GFF3 files.

#### Merging final results

GFF3 output from the previous *ab initio* and MetaEuk protein evidence steps were input into Gffread (106) (parameters: -G -merge) to localize predictions from both lines of evidence into a single GFF3 output file. Each locus was then merged together using a Python (107) script and the BioPython API (108) within EukMetaSanity. The set of *ab initio* generated exons in each locus is used as a prediction of the underlying exon/intron structure of the gene locus to which it is assigned. If there are any protein-evidence-generated exons present at the same locus, and if the total numbers of exons predicted by each line of evidence have ≥70% agreement, *ab initio* generated exons lacking a corresponding protein-evidence-generated exon are removed (the first and last exons of a locus are not removed). Conversely, any protein-evidence-generated exon present that lacks a corresponding *ab initio* generated exon is added to the predicted exon/intron structure. The final gene structure for each locus is then processed into GFF3 and FASTA format.

### Functional and taxonomic annotation of eukaryotic MAGs

Predicted proteins from EukMetaSanity were annotated for function against protein families in Pfam with PfamScan (109) and KEGG using kofamscan (110, 111) (Tables S7 and S8). The relative completeness and contamination of each putative Eukaryotic MAG was assessed based on protein content using BUSCO v 4.0.5 against the eukaryota_odb10 gene set using default parameters (26) and EukCC v 0.2 using the EukCC database [created 22 October 2019 (112)]. Annotation and completeness assessment were carried out using a EukHeist-Annotate (https://github.com/AlexanderLabWHOI/eukhesit-annotate). EukCC (112) was also used to calculate MAG completeness and contamination. The average completeness across groups increased in all cases with EukCC except for metazoans, which on average had a lower estimated completeness (Fig. S10).

The taxonomic affiliation of the high- and low-completion bins was estimated using MMSeqs taxonomy through EukMetaSanity and EUKulele (25), an annotation tool that takes a protein-consensus approach, leveraging a last common ancestor estimation of protein taxonomy, as well as MMSeqs2 taxonomy module (24, 95, 96). Taxonomic level estimation in EUKulele was assessed based on *e*-value derived best-hits, where percent id was used as a means of assessing taxonomic level, with the following cutoffs: species, >95%; genus, 95%–80%; family, 80%–65%; order, 65%–50%; class, 50%–30% modeled off of Carradec et al. (21). All MAGs were searched against the MarMetZoan combining the MarRef, MMETSP, and metazoan orthoDB databases (44, 97, 105, 113). MAGs with taxonomy assignment that did not resolve beyond SAR (Stramenopile, Alveolate, and Rhizaria) are classified as SAR. This database is available for download through EUKulele.

### Phylogeny of eukaryotic MAGs

A total of 49 BUSCO proteins were found to be present across 80% or more of the highly complete eukaryotic TOPAZ MAGs and were selected for the construction of the tree. Amino acid sequences from all genomes and transcriptomes of interest were collected and aligned individually using mafft (v7.471) (parameters: -thread -8 -auto) (114). Individual protein alignments were trimmed to remove sections of the alignment, which were poorly aligned with trimAl (v1.4.rev15) (parameters: -automated1) (115). Protein sequences were then concatenated and trimmed again with trimAl (parameters: -automated1). A final tree was then constructed using RAxML (v 8.2.12; parameters: raxmlHPC-PTHREADS-SSE3 -T 16 -f a -m PROTGAMMAJTT -N 100 -p 42 -x 42) (116). The amino acid alignment and construction were controlled with a Snakemake workflow: https://github.com/AlexanderLabWHOI/BUSCO-MAG-Phylogeny. Trees were visualized and finalized with Interactive Tree of Life (iTOL) (117).

### Prokaryotic MAG assessment and analysis

The 15,478 bins that were not identified as putative eukaryotic bins based on length and EukRep metrics were screened to identify quality prokaryotic bins. The quality and phylogenetic association of these bins were assessed with a modified version of MAGpy (118), which was altered to include taxonomic annotation with GTDB-TK v.0.3.2 (119). Bins were assessed based on single copy ortholog content with CheckM v (120) to identify two different bin quality sets: (i) high-quality prokaryotic bins (>90% completeness and <5% contamination) and (ii) medium-qualityprokaryotic bins (90%–75% completeness and <10% contamination). A total of 4,022 prokaryotic MAGs met the above criteria. A final set of 2,407 non-redundant HQ-MQ MAGs were identified using dRep v2.6.2 (121), which performs pairwise genome comparisons in two steps. First, a rapid primary algorithm, Mash v1.1.1 (122) is applied. Genomes with Mash values equivalent to 90% ANI or higher were then compared with MUMmer v3.23 (123). Genomes with ANI ≥ 99% were considered to belong to the same cluster. The best representative MAGs were selected based on the dRep default scoring equation (121). Out of the final set of 2,407 NR MAGs, 716 were HQ. The same pipeline was used to determine the HQ and MQ NR MAGs reconstructed from the Tara Oceans metagenomes in previous studies (11–13).

### Phylogeny of bacterial non-redundant high-quality MAGs

Only 5 out of the 716 HQ NR MAGs were found to belong to Archaea, thus only bacterial MAGs were used for the construction of the phylogenetic tree with GToTree v.1.4.10 (124) and the gene set (HMM file) for Bacteria (74 targets). GToTree pipeline uses Prodigal v2.6.3 (125) to retrieve the coding sequences in the genomes and HMMER3 v3.2.1 (126) to identify the target genes based on the provided HMM file. MUSCLE v3.8 (127) was then used for the gene alignments and Trimal v1.4 (15) for trimming. The concatenated aligned is used for the tree constructions using FastTree v2.1 (128). Three genomes were excluded from the analysis due to having too few of the target genes. The tree was visualized using the iToL (17).

### Prokaryote MAG phylogeny comparison

A set of 8,644 microbial genomes were collected from the MarDB database (113) (accessed 31 May 2018) encompassing the publicly available marine microbial genomes. Genomes were assessed using CheckM v1.1.1 (120) (parameters: lineage_wf) and genomes estimated to be <70% complete or >10% contamination were discarded. The remaining genomes (*n* = 5,878) were assessed using CompareM v0.0.23 (parameters: aai_wf; https://github.com/dparks1134/CompareM), and near identical genomes were identified using a cutoff of ≥95% average amino acid identity (AAI) with ≥85% orthologous fraction (determined as one standard deviation from the average orthologous fraction for genomes with 97–100% AAI). Based on CheckM quality, the genome with the highest completion and/or lowest contamination was retained. From the remaining genomes (*n* = 3,843), all MAGs derived from the Tara Oceans data set, specifically from Tully et al. (13) and Parks et al. (11), were removed. The remaining genomes (*n* = 2,275) would be used to form the base of a phylogenetic tree representing the available genome diversity prior to the release of previous Tara Oceans-related MAG data sets (11–13), termed the “neutral” component of subsequent phylogenetic trees.

For the comparisons, phylogenetic trees were constructed using GToTree v1.4.7 (124) (default parameters; 25 Bacteria_and_Archaea markers). Any genome added to a tree that did not meet the default 50% marker presence requirement was excluded from that tree. Five iterations of phylogenetic trees were constructed using the neutral genomes paired with each Tara Oceans MAG data set, the high-quality TOPAZ prokaryote MAGs, and the medium-quality TOPAZ prokaryote MAGs, individually, and two larger trees were constructed containing all neutral genomes and Tara Oceans MAGs, with additions of either high- or medium-quality TOPAZ MAGs. Phylogenetic trees were assessed using genometreetk (parameter: pd; https://github.com/dparks1134/GenomeTreeTk) to determine the phylogenetic diversity (i.e., the total branch length traversed by a set of leaves) and phylogenetic gain (i.e., the additional branch length added by a set of leaves) (11) for each set of MAGs compared against the neutral genomes and for the TOPAZ prokaryote MAGs compared against the neutral genomes and the other Tara Oceans MAGs.

### MAG abundance profiling

Raw reads from all metagenomic and metatranscriptomic samples were mapped against the eukaryotic and prokaryotic TOPAZ MAGs to estimate relative abundances with CoverM (v. 0.5.0; parameters: -min-read-percent-identity 0.95 -min-read-aligned-percent 0.75 -dereplicate -dereplication-ani 99 -dereplication-aligned-fraction 50 -dereplication-quality-formula dRep -output-format dense -min-covered-fraction 0 -contig-end-exclusion 75 -trim-min 0.05 -trim-max 0.95 -proper-pairs-only; https://github.com/wwood/CoverM). The total number of reads mapped to each MAG was then used to calculate reads per kilobase million (RPKM), where for some MAG, *i*, with *X* = total number of reads recruiting to a MAG, *l* = length of MAG in kb, and *N* = total number of trimmed reads mapping to a sample in millions. We also calculated counts per million (CPM), a normalization of the RPKM to the sum of all RPKMs in a sample. CPM, a modification of transcripts per million was first proposed by Wagner et al. (129) as an alternative to RPKM that reduces statistical bias. The metric has since been applied to metagenomics data, called genes per million (130). We chose not to more stringently cluster MAGs on the basis of genome content due to the documented utility of preserving population-specific genes (131); we show a comparison of the CoverM-based dereplication approach using fastANI to the dnadiff function of the MUMmer paper in Fig. S25.

### Nutritional modeling

To predict the trophic mode of the high-quality TOPAZ eukaryotic MAGs (*n* = 485), a Random Forest model (132) was constructed and calibrated using the ranger (133) and tuneRanger packages in R (134), respectively. The model was trained using KEGG Orthology annotations (110) from a manually curated reference trophic mode transcriptomic data set consisting of the MMETSP (44) and EukProt (59) (Data set S1 at https://osf.io/twz2f/). A total of 644 transcriptomes in this reference data set came from the MMETSP (44), after 22 transcriptomes were removed due to low coverage of KEGG and Pfam annotations (109). The remaining 266 came from the EukProt database (59), after 162 were removed due to having fewer than 500 present KOs. Nutritional strategy (phototrophy, heterotrophy, or mixotrophy) was assessed for each reference transcriptome individually based on the literature, 25% of the combined reference transcriptomes were excluded from model training as testing data (Fig. S16).

A subset of KOs that were predictive for trophic mode classification was determined computationally with the vita variable selection package in R (135) (Table S5), which has been tested and justified for this use case (136). This process was carried out by the algorithm without regard to the predicted function of the KOs, but we found that many of these KOs were implicated in carbohydrate and energy metabolism, with a preference for those KOs that differ strongly between heterotrophs and phototrophs (particularly for energy metabolism; Fig. S18). The model was built using the selected KOs (*n* = 1,787 of a total 21,585 KOs) with 75% of the combined database assigned as training data.

Additionally, we developed a secondary metric for assessing the extent of heterotrophy of a transcriptome or MAG. As opposed to the trinary classification scheme of the Random Forest model, this approach quantifies the extent which the MAG aligns with heterotrophic, phototrophic, or mixotrophic references by assigning a composite score. We calculated the likelihood of vita-selected KOs used in the Random Forest model above to be present within heterotrophic, phototrophic, or mixotrophic reference transcriptomes. Three scores (*h*, *p*, *m*), one corresponding to each trophic mode, were hence calculated for each vita-selected KO (*k*) (*n* = 1,787) (Table S5). In equation 1, *K* is the number of references the KO was present for each trophic mode category, while *n* is the total number of references available for each trophic mode category.


(1) $$\begin{array}{r} h_{k}=g\left(\frac{K_{\text{het}}}{n_{\text{het}}}\right) \end{array}$$

(2) $$\begin{array}{r} p_{k}=g\left(\frac{K_{\text{photo}}}{n_{\text{photo}}}\right) \end{array}$$

(3) $$\begin{array}{r} m_{k}=g\left(\frac{K_{\text{mixo}}}{n_{\text{mixo}}}\right) \end{array}$$

(4) $$\begin{array}{r} \text{where, }g(a)=\left\{ \begin{array}{ll} a & \text{if }a>0.5\\ -(0.5-a) & \text{ otherwise} \end{array}\right. \end{array}$$

If a given KO occurred in fewer than 50% of the reference transcriptomes for a trophic mode, it was considered not to be characteristic of that trophic mode and as such the score, which we represent as variable *a*, the ratio of the present KOs to the total for the subset of transcriptomes annotated some trophic mode (equation 4), was transformed [−(0.5 − *a*), if *a <* 0.5] to reflect the absence without valuing absence over presence. In the test transcriptome data set, the ratio-transformed scores were negated when a given KO was absent from the transcriptome. For instance, if a KO was absent from 90% of reference transcriptomes assigned to heterotrophy (*a* = 0.1) and absent in the MAG or transcriptome being evaluated, it would receive a score of *h_k_* = −1 × [−(0.5 − 0.1)] = 0.4 (equation 1) for that KO. This reflects that the absence of the KO in the evaluated MAG or transcriptome aligned well with the high probability that the KO was absent among the reference transcriptomes.

The scores for all KOs selected by vita were then used to scale the presence/absence patterns observed across transcriptomes and MAGs. Thus, for each transcriptome or MAG, a single score was calculated for each trophic mode heterotrophy (*H*), phototrophy (*P*), and mixotrophy (*M*) for all KOs present within the transcriptome or MAG (*K*)


(5) $$H=\sum_{k\in K}h_{k}\;$$

(6) $$P=\sum_{k\in K}p_{k}\;$$

(7) $$M=\sum_{k\in K}m_{k}$$

These calculated values can then be aggregated to a composite heterotrophy score (*H*_ind_) (Table S6). The score was computed as follows:


(8) $$H_{ind}=\left\{ \begin{array}{ll} -1^{\left(H-P\right)}\sqrt{(H-P{)}^{2}}, & \text{if}\;M-\max(H,P)<50,\\ \frac{-1^{\left(H-P\right)}\sqrt{(H-P{)}^{2}}}{M}, & \text{if}\;M-\max(H,P)\geq 50 \end{array}\right.$$

### Network analysis

To identify co-occurring MAGs across the stations surveyed by Tara Oceans, the CPM abundance of each highly complete eukaryotic MAG (>30% BUSCO completeness) and each non-redundant, highly complete bacterial MAG was assessed at each station at all available depths and size fractions as described above. CPM was used because of the power of this metric for comparing samples directly: the sum of all CPM values per sample will be the same, as sequencing depth is accounted for after gene length. This makes it easier to compare the abundances of MAGs originally recovered from different sites (130). A Spearman correlation matrix was generated to identify monotonic relationships between MAGs. Correlations were filtered based first on *P*-value, using the Šidák correction (137), a slightly less stringent metric than the Bonferroni correction. The Šidák correlation adjusts for multiple comparisons and is given by *P* < 1 − (1 − α)^1/*n*^, where *n* is the total number of comparisons and α is the significance value, in this case, 0.05. We considered only those correlations within the 90th percentile of CPM correlations, thus correlations with absolute value <0.504 were removed from the analysis. Subsequently, we further filtered interactions to those with a coefficient of correlation <0.70 for the construction of the network diagram. Because it was expected for several of the eukaryotic MAGs to be closely related (based on ANI), the relationships in the network were further filtered to exclude interactions between MAGs of exceedingly high similarity (having both 99% ANI similarity and >0.70 coefficient of correlation in the network analysis) (Table S8). ANI-based group members tended to have identical taxonomic classifications: only 2 of the 94 clusters had different classifications at the order level per EUKulele (Fig. S25).

We generated a network from this reduced set of labeled interactions (cut off at >0.70 coefficient of correlation, focusing on interactions between eukaryotes and prokaryotes or eukaryotes and eukaryotes, and using ANI-based clusters instead of MAG names when applicable) using igraph (138, 139) (Table S7). Communities of highly associated MAGs were identified using a modularity optimization algorithm introduced in (69) and implemented in igraph (138).

We assessed the connectedness within and between communities by calculating a connectedness metric as follows. For the connectedness within a community (one community to itself), we identified the number of “dense” connections by counting up the total number of links found between community members, regardless of how many times the particular MAG had been connected to its own community and divided that number by the total possible “dense,” meaning the number of connections which would exist if all community members were connected to all other community members. Between different communities, we defined connectedness by qualifying that a “connection” is made the first time each MAG from a given community is linked to another community and calculated this quantity by dividing the number of realized links between community members by the maximum total size of the two involved communities (Fig. 4b; equations 9-11).


(9) $$\begin{array}{r} C_{x,x}=\frac{\Sigma_{x=1}^{n_{x}}\Sigma_{y=1}^{n_{y}}f(x,x)}{\frac{n_{x}(n_{y-1})}{2}} \end{array}$$

(10) $$\begin{array}{r} C_{x,y}=\frac{\Sigma_{x=1}^{n_{x}}\Sigma_{y=x+1}^{n_{y}}f(x,y)}{\text{max}\;(n_{x},n_{y})} \end{array}$$

(11) $$f(a,b)=\left\{ \begin{array}{ll} 1 & if\;a\;and\;b\;are\;connected\\ 0 & otherwise \end{array}\right.$$

We calculated Spearman correlation coefficients for the relationship between the abundance of communities between stations and several environmental parameters of interest from the Tara Oceans metadata (70, 140) (Fig. 4). We considered the measured physical and chemical parameters, the modeled mesoscale physical oceanographic parameters, and averaged remote sensing products (70, 71, 140). We adjusted the *P*-value of these comparisons using a Bonferroni adjustment within the statistics package in R (139).

## Data Availability

The eukaryotic and prokaryotic TOPAZ MAGs and Tables S1 through S13 are available through the Open Science Framework (OSF) at https://osf.io/gm564/ with the DOI 10.17605/OSF.IO/GM564. EukHeist, which was used to recover the reported TOPAZ MAGs can be found at https://github.com/AlexanderLabWHOI/EukHeist, and EukMetaSanity, which was used for protein prediction in eukaryotic MAGs can be found at https://github.com/cjneely10/EukMetaSanity. The code used to generate the figures in this paper can be found at https://github.com/AlexanderLabWHOI/2021-TOPAZ-MAG-Figures. An interactive visualizer for the TOPAZ eukaryotic MAGs is available at https://taravisualize.streamlit.app/ with source code at https://github.com/cjneely10/TARA-Analysis.

## SUPPLEMENTAL MATERIAL

Supplemental Material mbio.01676-23-s0001.pdf Supplemental text, table captions, and figures.

Table S1 mbio.01676-23-s0002.csv Assembly group description, sample inclusion, and basic assembly statistics.

Table S2 mbio.01676-23-s0003.csv TOPAZ eukaryotic MAG taxonomy, genomic characteristics (e.g., total length, GC content, N50, number of predicted proteins), and estimated completeness and contamination.

Table S3 mbio.01676-23-s0004.csv TOPAZ prokaryotic MAG taxonomy as estimated by GTDB, data set indication (non-redundant representatives (NR), total size, and estimated completeness and contamination.

Table S4 mbio.01676-23-s0005.csv TOPAZ prokaryotic MAG summary of recovered MAGs across phyla.

Table S5 mbio.01676-23-s0006.csv Vita selected KOs and their associated heterotrophy, phototrophy, and mixotrophy ratios as described in equations 1-4.

Table S6 mbio.01676-23-s0007.csv Eukaryotic TOPAZ MAG predicted trophic status and heterotrophy index (H-index).

Table S7 mbio.01676-23-s0008.csv Network analysis community composition.

Table S8 mbio.01676-23-s0009.csv Eukaryotic cluster groups derived from average nucleotide identity clustering of eukaryotic TOPAZ MAGs with the Delmont eukaryotic MAGs (Delmont et al. 2022) based on an ANI cutoff of 99%.

Table S9 mbio.01676-23-s0010.csv Environmental correlations with network derived communities.

ASM does not own the copyrights to Supplemental Material that may be linked to, or accessed through, an article. The authors have granted ASM a non-exclusive, world-wide license to publish the Supplemental Material files. Please contact the corresponding author directly for reuse.
